# Health-related Quality of Life of Patients With Non–Intra-abdominal Desmoid-Type Fibromatosis During Active Surveillance: Results of a Prospective Observational Study

**DOI:** 10.1097/SLA.0000000000005795

**Published:** 2023-01-19

**Authors:** Anne-Rose W. Schut, Milea J.M. Timbergen, Kazem Nasserinejad, Thijs van Dalen, Winan J. van Houdt, Johannes J. Bonenkamp, Stefan Sleijfer, Dirk J. Grünhagen, Cornelis Verhoef, Olga Husson

**Affiliations:** * Department of Surgical Oncology, Erasmus MC Cancer Institute Rotterdam, The Netherlands; † Department of Medical Oncology, Erasmus MC Cancer Institute, Rotterdam, The Netherlands; ‡ Department of Hematology, Erasmus MC Cancer Institute, Rotterdam, The Netherlands; § Department of Surgical Oncology, University Medical Center Utrecht, the Netherlands; Diakonessenhuis Utrecht, The Netherlands; ∥ Department of Surgical Oncology, Netherlands Cancer Institute, Amsterdam, The Netherlands; ¶ Department of Surgical Oncology, Radboud University Medical Center, Nijmegen, The Netherlands; # Department of Medical Oncology, Netherlands Cancer Institute, Amsterdam, The Netherlands

**Keywords:** active surveillance, aggressive fibromatosis, desmoid tumor, health-related quality of life, patient-reported outcomes, wait-and-see

## Abstract

To examine the impact of an active surveillance (AS) approach on the health-related quality of life (HRQoL) of patients with desmoid-type fibromatosis (DTF).

**Background Data:**

AS is recommended as initial approach in DTF patients. AS might however negatively affect HRQoL due to physical symptoms or stress and anxiety.

**Methods:**

In a prospective observational study, the GRAFITI trial (NTR4714), DTF patients were followed during an initial AS approach for 3 years. HRQoL was assessed by the EORTC QLQ-C30 at baseline, 6, 12 and 24-month follow-up. Patients who completed questionnaires at≥1-time point were included in this analysis of the secondary endpoint. A multivariable linear mixed-effects model with random intercept was conducted to assess trends of HRQoL scores over time and to explore the effect of treatment strategy on HRQoL.

**Results:**

All 105 patients enrolled in the GRAFITI trial were eligible for the HRQoL analyses. During 24-month follow-up, 75 patients (71%) continued AS and 30 patients (29%) started an active treatment (AT). DTF patients who continued AS demonstrated relatively stable HRQoL scores during follow-up. HRQoL scores of patients who started AT worsened compared to patients who continued AS, although no significant changes in HRQoL score over time were found in the mixed-model analyses. Overall, DTF patients who started AT scored significantly worse on pain (β=10.08, *P*=0.039) compared to patients who continued AS.

**Conclusions:**

An initial AS approach did not impair HRQoL of DTF patients who continued AS over time, therefore providing further support for AS as the frontline approach in DTF patients. Longitudinal assessment of HRQoL should be part of clinical follow-up to identify patients who may need a change in treatment strategy.

Active surveillance (AS) is considered as the frontline approach in patients with desmoid-type fibromatosis (DTF), a rare, intermediate-grade soft tissue tumor with a highly variable clinical course.^1^–^4^ An initial AS approach is justified as DTF does not metastasize, has a high tendency to recur locally after surgery, and retrospective series reported long-lasting disease stabilization or spontaneous tumor regression without any treatment.^5^,^6^ AS is defined as continuous monitoring of DTF patients with imaging modalities, preferably Magnetic Resonance Imaging, at intervals of 3 to 6 months. Persistent radiological or symptomatic progression can necessitate the start of an active treatment (AT), such as surgical resection, systemic therapies, and local therapies.^1^ Two recent observational studies, one of which was the GRAFITI trial, provided the first prospective evidence supporting AS as an initial approach in DTF patients.^7^,^8^ Their results demonstrated that the majority of patients eventually developed a stable or regressive disease and that only 30% of the patients needed an active treatment. An initial AS approach thus minimizes overtreatment and avoids potential treatment-related morbidity.

However, an AS approach may affect the health-related quality of life (HRQoL) of DTF patients. HRQoL is a patient-reported outcome that includes the patients’ perception of his or her physical, emotional, cognitive, and social functioning.^9^ During AS, DTF patients can experience pain and physical symptoms caused by the tumor itself, even without demonstrating radiological tumor progression.^8^ Moreover, in other diseases than DTF, it has been reported that an AS approach can lead to increased stress and anxiety as a result of leaving a tumor untreated.^10^ Therefore, not only objective outcomes, such as radiological response, but also HRQoL outcomes are important to evaluate the effects associated with an AS approach.^3^ Here we present the HRQoL outcomes of DTF patients included in the GRAFITI trial, a prospective observational study in which patients were managed with an initial AS approach.

## METHODS

### Study Design and Population

The HRQoL data were collected within the GRAFITI trial, a prospective, multicentre observational study performed in 7 sarcoma centers in the Netherlands. The study was approved by the Ethics Committee of the Erasmus Medical Centre (MEC-2014-124), registered in the Dutch trial register (study ID: NTR4714), and all patients provided written informed consent. The study design, details on study procedures, and objective outcome measures of the GRAFITI trial have been published previously.^8^,^11^ Briefly, AS was evaluated as an initial approach for adult patients with non–intra-abdominal sporadic DTF without previous treatment for the current lesion. Patients with severe pain or functional impairment due to the tumor at the time of inclusion (as indicated by the patient) were excluded. The follow-up protocol consisted of follow-up visits and imaging examinations (ultrasound and MRI) during an AS approach for a minimum of 3 years. In case of tumor growth or progressive symptoms, the AS management strategy was re-evaluated according to the international guidelines.^1^ For these patients, the decision to start an AT was made by both the physician and the patient and was discussed in a multidisciplinary meeting. Active treatments could include systemic therapy, surgical resection, or radiotherapy. After the start of AT, clinical follow-up no longer took place in the GRAFITI trial setting. All patients were asked to complete the HRQoL questionnaire on paper at baseline and 6, 12, and 24 months after the start of an AS approach. After a switch to AT, patients were still asked to complete the HRQoL questionnaire. As per protocol, patients did not complete a questionnaire at 36-month follow-up, clinical and HRQoL data from baseline to 24-month follow-up were reported here. Progression-free survival and the cumulative incidence of the start of an active treatment at 3 years have been reported previously.^8^

### HRQoL Assessments

HRQoL was assessed by the European Organization for Research and Treatment of Cancer Quality of Life Questionnaire-Core 30 instrument (EORTC QLQ-C30).^12^ This 30-item HRQoL questionnaire consists of 5 functional scales (physical, role, cognitive, emotional, and social functioning), a global quality of life (QoL) scale, 3 symptom scales (fatigue, nausea, and vomiting, and pain) and 6 single items (appetite loss, diarrhea, dyspnea, constipation, insomnia, and financial difficulties) assessing common symptoms and perceived financial impact of the disease. The timeframe of the questions is the last week. Each item is scored on a Likert scale ranging from 1, “not at all” to 4, “very much”, with the exception of the global QoL scale, which is scored on a 7-point response scale ranging from 1, “very poor” to 7 “excellent”. Scores of all scales and single items are linearly transformed to a score between 0 and 100, according to the EORTC QLQ-C30 scoring manual.^13^ A scale score is generated if a patient answered at least half of the corresponding items of the scale. A higher score on the functional scales and global QoL means better functioning and HRQoL, whereas a higher score on the symptom scales means higher symptom burden.

### Statistical Analysis

All patients who completed questionnaires at≥1-time point were included in the HRQoL analyses. HRQoL scores of DTF patients included in the HRQoL analyses were presented as mean and SD. In addition, EORTC QLQ-C30 mean scores of the Dutch general population norms were presented (for males and females aged between 30–49 y).^14^ Other continuous variables were presented as the median and interquartile range. Categorical variables were described as numbers and percentages. Comparative analyses were performed with χ^2^ tests for categorical variables and Mann-Whitney *U* tests for continuous variables. A multivariable linear mixed-effects model with random intercept was used to analyze longitudinal changes in HRQoL. To explore the effect of the type of treatment strategy during follow-up on trends of HRQoL scores and overall HRQoL, a patient was assigned to 1 of the 2 ‘treatment groups’. The active surveillance (AS) group included patients who continued AS from baseline to 24-month follow-up. The active treatment (AT) group included patients who discontinued AS and started AT during 24 months of follow-up.

For each HRQoL subscale or single item of the EORTC QLQ-C30, the following multivariable model was employed.


$$HRQoL_{it}=\beta_{0}+b_{i}+\beta_{1}\;\;\times time_{it}\;+\beta_{2}\times group_{i}\;+\beta_{3}\times time_{it}\times group_{i}\;+\beta_{3}\times treatment\;status_{it}$$

Where ${\mathrm{HRQoL}}_{\mathrm{it}}$ indicates the HRQoL for the specified subscale or single item for the i^th^ patient at t^th^ time point, time was the follow-up [months], *group* was AS group versus AT group, treatment status was on AS versus on/after AT at the time a patient completed the questionnaire, β were the fixed parameters and $b_{i}$ was the random effects in the model. An interaction term between time and group was considered in the model to be able to explain the trend of HRQoL of both groups over time. No imputations were performed in case of missing data. Statistical analyses were performed using R version 3.6.1. (http://www.r-project.org/). For all analyses, 2-sided *P*<0.050 was considered statistically significant.

## RESULTS

### Patient Characteristics

Of the 105 patients enrolled in the GRAFITI trial, all 105 patients completed the EORTC QLQ-C30 questionnaire at least once and were thus eligible for the HRQoL analyses. Ninety-five patients (91%) completed the baseline questionnaire, 88 patients (84%) completed the 6-month questionnaire, 83 patients (79%) completed the 12-month questionnaire, and 75 patients (71%) completed the 24-month questionnaire. Baseline characteristics of the study population are summarized in Table 1 and published in detail previously.^8^ From baseline to 24-month follow-up, 75 patients (71%) continued AS and 30 patients (29%) discontinued AS and started some form of AT. The median time to AT was 9.2 months (interquartile range 5.5–16.0). Differences between the AS and AT groups at baseline were seen only in tumor size, with patients in the AT group having larger tumors. (*P*=0.007). At each follow-up visit, the proportion of patients completing the EORTC QLQ-C30 questionnaire was comparable between those completing the questionnaire during AS and those on/after AT (Supplemental Table 1, Supplemental Digital Content 1, http://links.lww.com/SLA/E414). Table 1.

**TABLE 1:** Baseline Characteristics of the HRQoL-analysis Study Population (N=105)

	Study population (N=105)	Active surveillance group* (n=71)	Active treatment group† (n=30)
Median age at time of diagnosis (y)	37 (32–47)	37 (33–49)	36 (31–46)
Sex, n (%)			
Male	21 (20)	14 (19)	7 (23)
Female	84 (80)	61 (81)	23 (77)
Tumor localization, n (%)			
Abdominal wall	37 (35)	30 (40)	7 (23)
Head and neck	8 (8)	3 (4)	5 (17)
Upper extremity	7 (7)	4 (5)	3 (10)
Trunk and back	25 (24)	19 (26)	6 (20)
Breast	10 (9)	7 (9)	3 (10)
Lower extremity	18 (17)	12 (16)	6 (20)
Recurrent disease, n (%)			
Yes	6 (6)	4 (5)	2 (7)
Median tumor size (cm)	4.1 (3.0–6.6)	3.8 (3.0–6.0)	5.0 (3.8–7.6)
Symptoms at time of inclusion‡, n (%)			
Yes	68 (65)	49 (65)	19 (63)
Mean HRQoL scores (*n*=95)§			
Functioning scales¶			
Global health	72.6 (19.4)	72.0 (19.8)	74.1 (18.5)
Physical functioning	88.9 (14.8)	88.2 (15.8)	90.6 (12.3)
Role functioning	81.4 (23.0)	82.1 (23.6)	79.6 (21.8)
Emotional functioning	72.1 (25.5)	71.9 (24.9)	72.5 (27.6)
Cognitive functioning	86.5 (19.4)	86.3 (20.1)	87.0 (18.1)
Social functioning	85.6 (22.3)	85.6 (21.9)	85.8 (23.9)
Symptom scales/items||			
Fatigue	23.5 (23.4)	23.2 (23.7)	24.3 (23.1)
Nausea and vomiting	4.4 (11.2)	4.9 (11.9)	3.1 (9.3)
Pain	22.8 (22.9)	21.1 (22.6)	27.2 (23.6)
Dyspnea	7.4 (18.3)	7.4 (17.1)	7.4 (21.4)
Insomnia	20.7 (27.1)	18.1 (26.7)	27.2 (27.8)
Appetite loss	5.6 (14.3)	4.9 (13.2)	7.4 (16.9)
Constipation	7.4 (17.7)	6.0 (14.1)	11.1 (24.5)
Diarrhea	11.9 (39.8)	12.1 (44.8)	11.1 (24.5)
Financial difficulties	7.8 (20.4)	9.5 (22.3)	3.7 (14.1)

Values are presented as median (IQR), mean (±SD) or n (%).

* Patients who continued active surveillance from baseline to 24-month follow-up.

† Patients who started an active treatment during 24-month follow-up.

‡ Sensory symptoms, motoric symptoms, cosmetic complaints, pain, cramps.

§ Mean HRQoL scores were calculated based on the available questionnaires at baseline.

¶ Higher scores indicate better functioning.

|| Higher scores indicate a higher level of symptomatology / problems.

HRQoL indicates health-related quality of life; IQR, interquartile range.

### Changes in HRQoL Over Time

**FIGURE 1:**
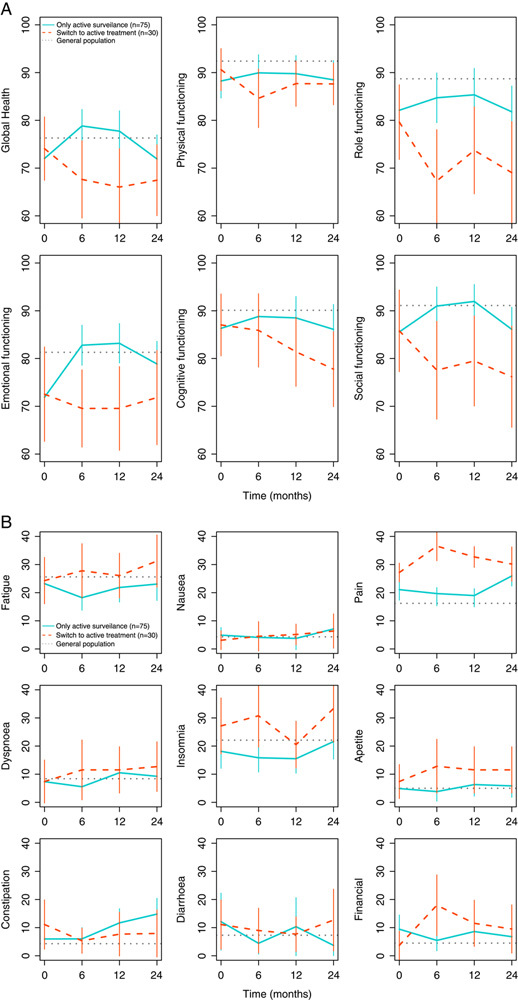
A and B. HRQoL scores from baseline to 24-month follow-up by treatment group for all EORTC QLQ-C30 functioning scales and global health scale (A) and symptom scales and items (B). HRQoL scores of the treatment groups are reported as mean score and 95% confidence interval. The horizontal line presents the mean HRQoL score of the Dutch general population norm data based on Nolte et al^14^ of males and females aged 30 to 49 years (n=324). A higher score on the functional scales and global quality of life means better functioning and global health, whereas a higher score on the symptom scales means higher symptom burden. HRQoL indicates health-related quality of life.

Figure 1 presents the mean HRQoL scores of DTF patients who continued AS and of patients who started AT during 24-month follow-up for all EORTC QLQ-C30 subscales and single items. The visual presentation showed that the mean HRQoL scores of patients who continued AS remained relatively stable. HRQoL scores in the AS were comparable to the mean scores of the Dutch general population norms,^14^ except for physical functioning, role functioning, insomnia, and pain. Compared with HRQoL scores of patients who started AT, DTF patients who continued AS scored better (ie, higher scores on functioning scales and lower scores on symptom scales and items) on all functioning scales and symptom items related to fatigue, pain, insomnia, and appetite loss. Figures 1A and B.

The results of the multivariate analyses are presented in Table 2. In general, no significant trends of HRQoL scores over time were observed for the AS group. Trends of HRQoL scores over time for the AT group did not differ significantly from the AS group, except for the cognitive functioning scale (β=−0.58, *P*=0.022), with worse cognitive functioning over time for DTF patients in the AT group. Overall, DTF patients who started AT during 24-month follow-up had significantly higher scores, indicating more problems, on the symptom scale related to pain (β=10.08, *P*=0.039), compared with those who continued AS. Most HRQoL scores of DTF patients in the AT group did not change after AT was started compared to their HRQoL scores while they were still on AS; only HRQoL scores on physical functioning were significantly worse after treatment change (β=−5.72, *P*=0.046) Table 2.

**TABLE 2:** Multivariable mixed-model Analyses of Changes in HRQoL scores for all DTF Patients (*N*=105) and for all EORTC QLQ-C30 Subscales

	Multivariable model							
	Time		Time× treatment during follow-up‡ (interaction)		Treatment during follow-up‡		Treatment status§	
	Change rate (β)	*P*	Change rate (β)	*P*	Change rate (β)	*P*	Change rate (β)	*P*
Functioning scales*								
Global health	−0.09	0.426	−0.30	0.293	−4.07	0.297	3.22	0.523
Physical functioning	0.03	0.593	0.01	0.962	−0.41	0.903	−5.73	**0.046**
Role functioning	0.01	0.894	−0.03	0.925	−7.02	0.167	−6.68	0.211
Emotional functioning	0.19	0.119	−0.37	0.234	−5.41	0.119	5.07	0.370
Cognitive functioning	−0.06	0.550	−0.58	**0.022**	−1.13	0.786	5.78	0.208
Social functioning	0.04	0.667	−0.37	0.160	−3.70	0.404	−0.16	0.974
Symptom scales/items†								
Fatigue	0.09	0.410	0.17	0.560	2.72	0.580	2.84	0.588
Nausea	0.06	0.426	−0.05	0.789	−1.09	0.647	2.90	0.410
Pain	0.16	0.155	0.17	0.566	10.08	**0.039**	−3.39	0.518
Dyspnea	0.12	0.204	−0.26	0.299	2.50	0.566	6.71	0.145
Insomnia	0.17	0.243	0.03	0.934	9.93	0.057	−1.53	0.822
Appetite loss	0.09	0.386	−0.19	0.430	4.98	0.169	3.59	0.411
Constipation	0.33	**0.002**	−0.41	0.151	1.93	0.635	0.25	0.961
Diarrhea	−0.28	0.222	0.28	0.617	−0.97	0.863	2.05	0.826
Financial difficulties	−0.07	0.520	0.18	0.542	0.39	0.926	2.90	0.579

Bold indicates significance (*P*<0.050).

* Higher scores indicate better functioning.

† Higher scores indicate a higher level of symptomatology / problems.

‡ Patients who continued active surveillance (AS) during from baseline to 24-month follow-up (AS group) versus patients who started an active treatment (AT) during 24-month follow-up (AT group).

§ Patients who were on AS versus patients who were on/after an AT at the time a patient completed the questionnaire.

AS indicates active surveillance; AT, active treatment.

## DISCUSSION

In the GRAFITI trial, patients with non–intra-abdominal DTF were prospectively observed during an initial AS approach. The current study shows that an AS approach did not negatively affect the HRQoL of DTF patients who continued AS. Overall, patients who needed AT showed worse HRQoL scores during follow-up compared with patients who continued AS.

Since the mortality rate of DTF is low, AS is considered to be the frontline approach, and active treatment options do not guarantee tumor reduction or clinical benefit, DTF has become a chronic condition for a significant proportion of patients.^1^,^5^,^15^–^17^ Therefore, when evaluating treatment effectiveness, not only objective outcomes should be taken into account, but also the impact on the HRQoL. It has previously been demonstrated that AS is a safe and effective management strategy for DTF patients in terms of progression-free survival and treatment-free survival.^7^,^8^ To the best of our knowledge, this is the first study reporting longitudinal HRQoL outcomes of DTF patients during AS.

In the current study, DTF patients who continued AS demonstrated relatively stable HRQoL scores during follow-up, and their HRQoL scores were comparable with the Dutch general population norms. Physical functioning and role functioning of DTF patients who were on AS were slightly worse, which is in line with the results of a study by Timbergen et al.^17^ Reduced physical functioning may be explained by functional limitations or pain caused by the DTF tumor, as the pain scores of patients in the AS group were slightly increased. Reduced physical functioning and pain may subsequently be 1 of the reasons for reduced role functioning, as patients may experience more discomfort in their daily activities. However, most differences in mean HRQoL scores between DTF patients who continued AS and the general population were small and HRQoL scores of patients who were under AS did not significantly change during follow-up. Only the symptom item constipation worsened significantly during follow-up in the AS group. Since the difference in mean scores was only minimal with a small β-value and the fact that patients with intra-abdominal DTF were excluded from the GRAFITI trial, this was considered as a coincident finding. Emotional functioning did seem to be impaired at the time the decision for an AS approach was made and patients were included in the GRAFITI trial, but it improved during follow-up for DTF patients who continued AS. The emotional functioning scale of the EORTC QLQ-C30 includes items related to worries, stress, and mood. Since not actively treating a disease, along with the unpredictable clinical course of DTF, can cause uncertainties, anxiety, and stress, it is not entirely unexpected that emotional functioning is affected in these patients.^10^,^17^–^19^ In a cross-sectional study that evaluated HRQoL among different groups of DTF patients by using a DTF-specific questionnaire (DTF-QoL), patients who received only AS also reported problems related to concerns about their condition, the unpredictable clinical course of DTF, and the emotional and psychological consequences of DTF.^20^ Studies on HRQoL of patients with low-risk prostate cancer on AS found that anxiety and uncertainty play a particular role at the moment of treatment choice and lead patients to either not choose or stop an AS approach quickly, which could also explain the improvement after the start of AS and subsequent stabilization of emotional functioning in DTF patients in our study.^18^,^21^ This emphasizes the importance of making patients understand the natural behavior of DTF during the treatment decision-making process and why AS is the frontline approach in DTF, as an AS approach may seem obvious to physicians but highly unreasonable to patients.^18^

During the follow-up of the GRAFITI trial, AT was started when AS was no longer feasible due to tumor progression and/or symptomatic progression alone. DTF patients who started AT had significantly larger tumors at baseline compared with patients in the AS group. The association between tumor size and the risk of starting AT has been described previously.^8^ Compared with patients who continued AS, DTF patients who needed an AT demonstrated worse HRQoL scores on all functioning scales, with a decrease in the cognitive, role, and social functioning during follow-up. The need for AT or AT itself thus had a particular impact on how patients perceived others’ understanding of their condition and their ability to carry out their daily activities. This may be explained by the fact that these patients suffered more from physical symptoms, such as insomnia, fatigue, and pain, which made functioning more difficult. Interestingly, HRQoL scores on physical functioning did not differ much from the AS group. However, it could be that DTF-specific issues related to physical functioning were missed by using only the EORTC QLQ-C30.^20^ Although the differences in mean HRQoL between the AS and AT groups were evident at visual presentation, no significant differences were seen between the groups in the multivariate analysis. This may be explained by the relatively low number of patients who needed AT (n=30), which resulted in wide CIs and may have led to insufficient power to find significant trends. The number of patients who completed a questionnaire after the start of an AT is even lower, which makes it difficult to make reliable statements about the effect of AT on the HRQoL. Most of the HRQoL scores seem to stabilize or improve slightly at the end, which could indicate a benefit of the AT. On the contrary, HRQoL scores on physical functioning were significantly lower after AT was started than before treatment, which could be a result of treatment-related morbidities. Future studies, including larger numbers and longer follow-ups are needed to further evaluate the effect of the switch from AS to AT on HRQoL.

There are limitations to the present study. Firstly, the type and number of treatments DTF patients in the AT group received, which is also related to the complexity of the tumor, could have had an impact on HRQoL.^20^ However, details on the number and duration of treatments are lacking since follow-up in the GRAFITI trial was stopped after a patient started AT. Due to the relatively low number of patients who started AT, the HRQoL scores of the different treatment types could not be compared. Secondly, HRQoL of DTF patients might also have been influenced by tumor location, comorbidities, personal circumstances, and other socio-demographic and clinical variables, which were not corrected for. Finally, the lack of the use of a DTF-specific HRQoL questionnaire may have prevented the recognition of DTF-specific issues related to AS during follow-up, possibly leading to an overestimation of HRQoL.^22^,^23^ However, the use of a generic questionnaire as the EORTC QLQ-C30 did allow comparison with the general population.

After an initial AS approach, a switch to AT might be indicated for DTF patients with large, progressive and/or highly symptomatic tumors.^1^,^3^ The observation that DTF patients who needed an AT in this prospective study showed a decrease in their HRQoL scores, compared with relatively stable scores of patients who could continue an AS approach, indicates that HRQoL outcome measures might be useful alongside objective outcome measures to identify DTF patients for whom AT may be more appropriate. In addition to the EORTC QLQ-C30, DTF-specific HRQoL scores related to pain and physical limitations, for example, the ‘physical consequences’ and ‘pain and discomfort’ symptom scales of the DTF-QoL, could help identify which patients may need a change in treatment strategy or additional supportive care, for example, pain management. Adequate pain control as a first step may prevent the need to switch to more aggressive antitumor treatments.^3^,^8^ The ‘concerns about condition’, ‘unpredictable course and nature of DTF’ and ‘emotional and psychological consequences’ subscales of the DTF-QoL could be used in clinical follow-up to evaluate the psychosocial impact of an AS approach and could provide insight into which patients require additional psychological support during AS. In addition, knowledge of the risk factors for failure of an AS approach and for impaired HRQoL will help to provide better educational care at the time of treatment choice and better supportive care during clinical follow-up.^8^,^20^

To conclude, an initial AS approach does not impair the HRQoL of DTF patients who continue with AS over time. This study, therefore, provides further support for AS as the frontline approach in DTF patients. Longitudinal assessment of generic and DTF-specific HRQoL outcomes should be part of clinical follow-up of DTF patients to evaluate treatment efficacy and to facilitate shared decision-making between AS and AT.

## Supplementary Material
