# Supplementary material for: Health-related Quality of Life of Patients With Non–Intra-abdominal Desmoid-Type Fibromatosis During Active Surveillance: Results of a Prospective Observational Study
Source: Ann Surg. 2023 Jan 19;277(6):877–83. doi: 10.1097/SLA.0000000000005795 (PMC10174102; doi:10.1097/SLA.0000000000005795)
Supplement: Supplementary file 1 [file sla-277-00877-s001.pdf]

Supplemental Table 1. Number of Desmoid-type Fibromatosis patients who were under active surveillance or on/after active treatment and who completed the EORTC QLQ-C30 questionnaire per follow-up visit.

n (%), percentage of total patients on active surveillance or on/after active treatment at specific follow-up visit

		Number of patients	
Follow-up visit		On active surveillance	On/after active treatment
Baseline	Total	105	-
	Completed	95 (91%)	-
6 months	Total	99	6
	Completed	83 (84%)	5 (83%)
12 months	Total	86	19
	Completed	68 (79%)	15 (79%)
24 months	Total	75	30
	Completed	54 (72%)	21 (70%)
